# The mRNA-bound proteome of the early fly embryo

**DOI:** 10.1101/gr.200386.115

**Published:** 2016-07

**Authors:** Hans-Hermann Wessels, Koshi Imami, Alexander G. Baltz, Marcin Kolinski, Anastasia Beldovskaya, Matthias Selbach, Stephen Small, Uwe Ohler, Markus Landthaler

**Affiliations:** 1 Berlin Institute for Medical Systems Biology, Max-Delbrück-Center for Molecular Medicine, 13125 Berlin, Germany;; 2 Department of Biology, New York University, New York, New York 10003, USA;; 3 Department of Biology, Humboldt University, 10115 Berlin, Germany

## Abstract

Early embryogenesis is characterized by the maternal to zygotic transition (MZT), in which maternally deposited messenger RNAs are degraded while zygotic transcription begins. Before the MZT, post-transcriptional gene regulation by RNA-binding proteins (RBPs) is the dominant force in embryo patterning. We used two mRNA interactome capture methods to identify RBPs bound to polyadenylated transcripts within the first 2 h of *Drosophila melanogaster* embryogenesis. We identified a high-confidence set of 476 putative RBPs and confirmed RNA-binding activities for most of 24 tested candidates. Most proteins in the interactome are known RBPs or harbor canonical RBP features, but 99 exhibited previously uncharacterized RNA-binding activity. mRNA-bound RBPs and TFs exhibit distinct expression dynamics, in which the newly identified RBPs dominate the first 2 h of embryonic development. Integrating our resource with in situ hybridization data from existing databases showed that mRNAs encoding RBPs are enriched in posterior regions of the early embryo, suggesting their general importance in posterior patterning and germ cell maturation.

Post-transcriptional regulatory mechanisms play crucial roles in a wide variety of biological processes (Bartel 2009; Gerstberger et al. 2014; Mitchell and Parker 2014). In particular, system-wide studies in invertebrates and vertebrates showed that post-transcriptional regulation is critical for early development (Hamatani et al. 2004; Lécuyer et al. 2007; Qin et al. 2007; Bushati et al. 2008; Thomsen et al. 2010; Bazzini et al. 2012). In early stages, the zygotic genome is transcriptionally silent, and development is guided by maternally produced mRNAs and proteins that are loaded into the oocyte (Lasko 2011).

Oogenesis and early embryogenesis in *Drosophila* rely on mRNA localization, translational control, and the coupling of these processes (Lasko 2011; Laver et al. 2015). For example, the mRNA produced by the maternal effect gene *oskar* is translationally repressed during production in the nurse cells and transport into the oocyte. Repression of *oskar* translation is mediated by the RNA-binding protein (RBP) Bruno (Kim-Ha et al. 1995; Castagnetti et al. 2000). This repression is relieved only after localization of the *oskar* mRNA to the posterior-most region of the embryo. Translation of *oskar* requires *cis*-acting RNA elements in its 3′ UTR (Kim-Ha et al. 1993), deposition of the exon junction complex onto spliced *oskar* mRNA (Hachet and Ephrussi 2004; Palacios et al. 2004), and binding of the RBP Staufen to the *oskar* transcript (Ephrussi et al. 1991; Kim-Ha et al. 1991; Ferrandon et al. 1994). Oskar directs the posterior localization of a second mRNA (*nanos*), enhancing its translation in the germ plasm by preventing de-adenylation (Ephrussi et al. 1991; Zaessinger et al. 2006). Nanos protein also binds mRNA and is required for primordial germ cell differentiation and for posterior body patterning (Lehmann and Nüsslein-Volhard 1991; Gavis and Lehmann 1992; Kobayashi et al. 1996). Thus, an intricate network of RBPs establishes embryo polarity, setting the stage for transcriptional mechanisms that segment the embryo and position morphological structures along the embryo body plan.

Many *Drosophila* RBPs, including Oskar, Staufen, and Nanos, were identified through genetic screens, but a complete understanding of the role of RNA in embryogenesis require comprehensive, transcriptome-wide methods. Previous studies have described in vitro approaches for discovering large numbers of RBPs, including screening protein arrays for RNA-binding activities (Scherrer et al. 2010; Tsvetanova et al. 2010) and RNA affinity chromatography of cellular extracts followed by mass spectrometry (Tsvetanova et al. 2010; Dürnberger et al. 2013). In vivo, UV crosslinking has been used to capture physiological protein-mRNA interactions, which are then purified by oligo(dT) affinity chromatography and analyzed by mass spectrometry. These methods have been used in yeast, HeLa and HEK293 cells, and mouse embryonic stem cells (Baltz et al. 2012; Castello et al. 2012; Kwon et al. 2013; Mitchell et al. 2013) and have identified hundreds of RBPs with canonical and noncanonical RNA-binding domains (RBDs).

## Results

### In vivo mRBPome capture in fly embryo

**Figure 1.**
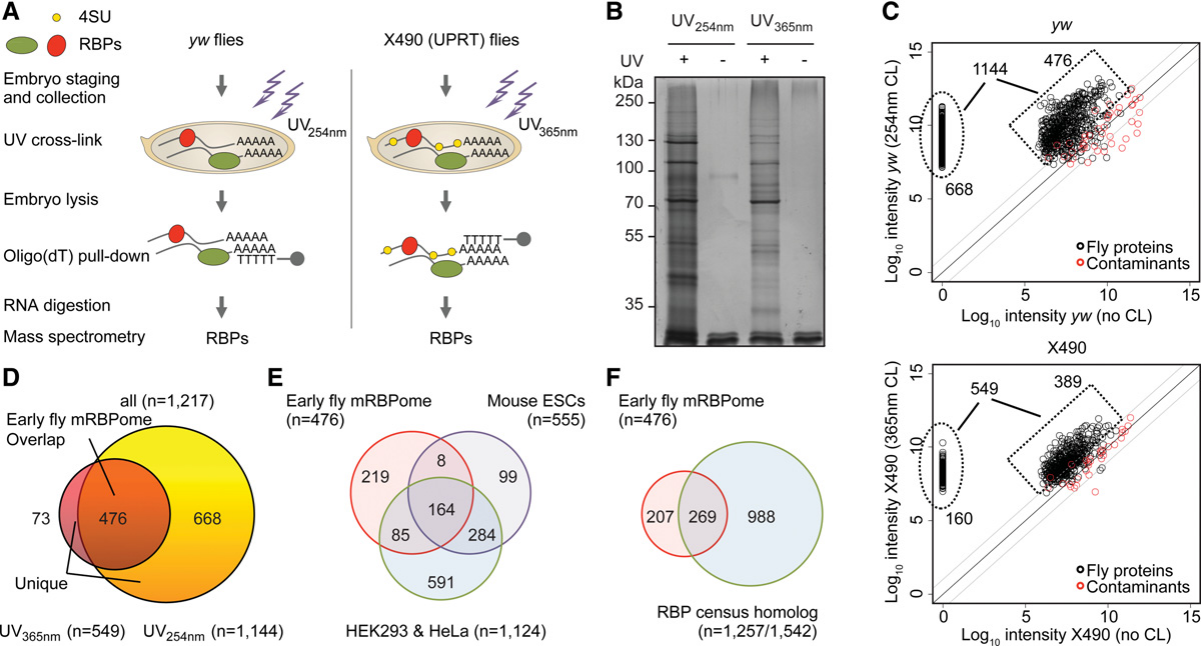
Identification of poly(A)^+^ RNA-bound proteins in fly embryos. (*A*) Schematic overview of mRNA interactome capture in early *yw* and X490 *Drosophila melanogaster* embryos. (*B*) Oligo(dT) precipitates of UV-crosslinked (+) and noncrosslinked (−) lysed *yw* and X490 embryos were separated by SDS-PAGE and silver-stained. (*C*) Scatterplot showing log_10_-transformed intensities of proteins in oligo(dT) pull-downs from crosslinked embryos versus log_10_-transformed intensities of proteins in oligo(dT) precipitates from noncrosslinked *yw* (*top*) and X490 (*bottom*) embryos. Proteins with at least two unique peptides and greater than 10-fold intensity in oligo(dT) pull-downs from crosslinked embryos compared to oligo(dT) precipitates from noncrosslinked embryos are considered as enriched and part of the early fly RNA-bound proteome (black). Common contaminant proteins (e.g., trypsin and keratin) are marked in red. (*D*) Venn diagram depicting the overlap of RNA-bound proteins (*n* = 1217) identified in *yw* (*n* = 1144) and/or X490 (*n* = 549) embryos. Four hundred seventy-six proteins overlap in both approaches and are referred to as the early fly mRBPome. Seven hundred forty-one proteins were uniquely identified. (*E*) Overlap of the early fly mRBPome (*n* = 476) to mRNA interactome studies in mouse mESCs (Kwon et al. 2013) and human HEK 293 and HeLa cells (Baltz et al. 2012; Castello et al. 2012) and (*F*) to the human RBP census (Gerstberger et al. 2014).

To identify poly(A)^+^ RNA-bound proteins in prezygotic and early zygotic *Drosophila melanogaster* embryos, we performed mRNA interactome capture and UV-crosslinking in 0–2-h old embryos of wild-type *yw* and X490 flies using conventional 254-nm (cCL) or photoactivatable-ribonucleoside-enhanced 365-nm UV-crosslinking (PAR-CL), respectively (Fig. 1A; Baltz et al. 2012; Castello et al. 2012). Incorporation of photoreactive 4-thiouridine (4SU) into nascent RNA was facilitated by TU-tagging using maternally expressed uracil phosphoribosyltransferase (*UPRT*) (Miller et al. 2009) (see Supplemental Methods). Adult females expressing *UPRT* during oogenesis consumed food with 4-thiouracil (4TU), which becomes converted into 4SU and incorporated into nascent RNA, specifically in nurse cells. This leads to efficient incorporation of 4SU into maternal mRNA and allows for PAR-CL in early embryos (Supplemental Fig. S1A).

UV-crosslinking greatly enhanced protein recovery in poly(A)^+^ RNA precipitates (Fig. 1B), with a total of 2013 proteins identified by mass spectrometry. And precipitated proteins were remarkably different from a control whole-embryo proteome (Supplemental Fig. S1B). Proteins derived from the UV-crosslinked embryos mostly exhibited higher intensities than proteins in precipitates of noncrosslinked embryos (Fig. 1B,C), whereas common contaminant proteins (e.g., trypsin and keratins) showed similar intensities in both preparations (Fig. 1C). UV-irradiation allowed specifically for enrichment of an RNA-interacting protein, AGO1, and not DNA-binders like Histone 3 (Supplemental Fig. S1C). Both UV-crosslinking approaches led to similar protein enrichment in the precipitates of *yw* and X490 flies (*r* = 0.77) (Supplemental Fig. S1D), and the identification was independent of embryonic protein abundance (Supplemental Fig. S1E). The embryonic proteome of *yw* and X490 flies correlated well (Supplemental Fig. S1F), and lengths of identified proteins were similar in precipitates and input samples (Supplemental Fig. S1G).

To define a stringent set of RBPs, we considered (1) proteins with at least 10-fold higher intensity in the oligo(dT) precipitates from crosslinked embryos when compared to oligo(dT) pull-downs from noncrosslinked embryos, and (2) proteins identified by at least two unique peptides in crosslinked embryos. One thousand two hundred seventeen proteins met both these criteria (549 proteins in PAR-CL, 1144 proteins in cCL); 476 proteins were detected by both mRNA interactome capture approaches (referred to as the “early fly mRBPome”), and 741 were uniquely identified (referred to as “unique set”) (Fig. 1C,D).

### Comparison with previous RNA interactome studies and the human RBP census

mRNA interactome capture has been used for human (HEK293 *n* = 797, HeLa *n* = 865) and mouse (mESC *n* = 555) cell lines (Baltz et al. 2012; Castello et al. 2012; Kwon et al. 2013), as well as yeast (*n* = 120) (Mitchell et al. 2013). In addition, a census of 1542 human RBPs (with 1257 orthologous proteins in fly) has been curated recently (Gerstberger et al. 2014). Comparing the early fly mRBPome with these protein sets, we identified 257 fly proteins with orthologs in mouse and/or human cells, and 269 overlapping to the human RBP census (Fig. 1E,F; Supplemental Fig. S1H).

The fly mRBPome contains 164 proteins that overlap all three mammalian mRNA capture studies, suggesting a conserved ‘core’ animal mRBPome. This core set is enriched for general RBP functions, e.g., translation, RNA processing, and mRNA metabolic process (Supplemental Table S1). In contrast, the set of fly-specific proteins were enriched for post-transcriptional gene silencing, RNA localization, microtubule cytoskeleton organization, and oogenesis (Supplemental Table S1). Forty-seven fly-specific proteins, including Exu, Vas, and Oskar, do not have any human or mouse ortholog.

### Characteristics of the early fly mRBPome

**Figure 2.**
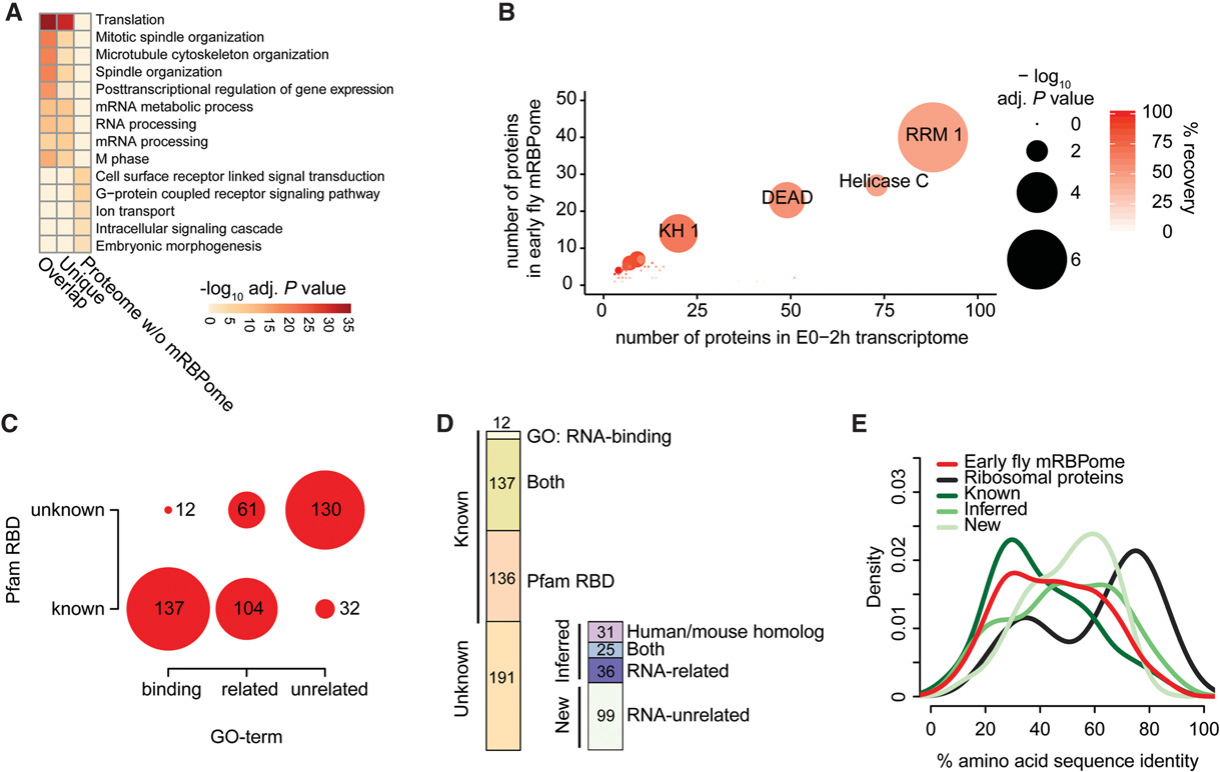
Characterization of the early fly mRBPome. (*A*) GO analysis showing the five most enriched gene ontology terms for molecular functions (GOMF) of the mRNA-bound proteins (overlap and unique) and the remaining proteins identified from whole embryos. *P* values were calculated by comparing against the early embryo transcriptome (0–2-h old embryos, all genes with FPKM > 0), adjusted for multiple testing with Benjamini-Hochberg and −log_10_-transformed. (*B*) Pfam protein domain enrichment of the early embryo mRBPome (*n* = 476) (*y*-axis) compared to the early embryo transcriptome (*n* = 7298) (*x*-axis). *P* values were calculated with Fisher's exact test and Bonferroni-corrected for multiple testing, indicated by circle size. Red shading indicates recovery percentages of expressed genes. (*C*) The intersection of proteins with known RNA-binding GO-term and Pfam RNA-binding domain (RBD) (list of RBDs and RNA-binding GO-terms described by Gerstberger et al. [2014]). (*D*) Proportions of proteins previously annotated as known RBPs by either GO-term or RBD (Known) and proteins not previously found to directly interact with RNA (Unknown). Unknown proteins contain homologs identified in mouse and human mRNA interactome studies and/or are part of the human RBP census, and proteins with RNA-related GO-terms (together referred to as ‘inferred RBPs’). ‘New’ refers to the 99 proteins undescribed in terms of RNA-binding. (*E*) Protein amino acid sequence identity to human homologous proteins using Ensembl Compara (Vilella et al. 2009). The early fly mRBPome without ribosomal proteins (*n* = 371) is depicted in subgroups containing: ribosomal proteins (*n* = 56), all known RBPs (*n* = 206), inferred RBPs (*n* = 80), and new RBPs (*n* = 85).

Using Gene Ontology (GO) terms and relevant domain annotations by Pfam as proxy to define known RBPs, we observed that RNA-binding domains and RNA-associated GO-terms are enriched in both the early fly mRBPome and the uniquely identified proteins (Fig. 2A). The remaining fraction of the total early fly proteome was depleted for such terms. The most enriched protein domains were known RBDs such as RRM, KH, and DEAD domains (Fig. 2B; Supplemental Fig. S2A,B). From a catalog of 799 RBDs encoded by 1063 fly genes expressed in the first 2 h of embryogenesis, we recovered 562 proteins in our total crosslinked set, similarly enriching for abundant canonical and less abundant noncanonical RBDs (Supplemental Fig. S2A, middle).

Two hundred seventy-three (57%) proteins in the early fly mRBPome contain at least one RBD (Fig. 2C; Supplemental Fig. S2C). Thirty-two of the RBD-containing proteins are not annotated as RNA-binding or with RNA-related functions. While 137 RBD-harboring proteins are considered as RNA-binding by GO, an additional 12 RNA-binding annotated proteins are missing a known RBD. In total, 285 of the 476 proteins are known RBPs in *Drosophila melanogaster* (Fig. 2D). Another 56 proteins are homologous to human and mouse RBPs and thus likely possess RNA-binding activity (Fig. 1E,F). Of the remaining 135 proteins, 36 are annotated to have RNA-related functions, whereas 99 proteins have not been previously implicated in RNA-related processes. We refer to homolog RBPs and RNA-related RBPs as ‘inferred’ set, while the 99 undescribed proteins are referred to as ‘new.’

Proteins missed in our approach were zinc-finger domain-containing gene products (e.g., ZF.C2H2 or ZF.MET domains) (Supplemental Fig. S2A,B), which are typically considered to be DNA-binders (Vaquerizas et al. 2009). On the other hand, we detected domains like Helicase C, WD40, and protein kinase domains, which are not considered classic RBDs (Supplemental Fig. S2B) or in case of the latter, may not interact themselves with RNA.

The degree of similarity between fly and human homologs ranged from 30%–70% amino acid sequence identity (Supplemental Fig. S2D, top). As previously described (Anantharaman et al. 2002), RBPs show higher levels of conservation than transcription factors, which may be more recently derived (Supplemental Fig. S2D, bottom). Interestingly, proteins we considered to be inferred or new RBPs exhibited even slightly higher conservation to human than known RBPs (Fig. 2E).

RBPs display more basic isoelectric points (pI) and an enrichment of disordered protein regions (Castello et al. 2012; Kwon et al. 2013). Accordingly, the early fly mRBPome exhibited an overall shift toward higher pI relative to the total early embryo proteome (Supplemental Fig. S2E). The mRBPome also showed a higher proportion of amino acids present in disordered and low complexity regions (Supplemental Fig. S2F,G) and are characterized by higher proportions of arginines, lysines, and glycines (Supplemental Fig. S2H). These trends were more pronounced in RBD-containing RBPs (Supplemental Fig. S2E–G, bottom).

RNA granules are rich in RBPs (Kato et al. 2012), and 143 of 476 proteins in the early fly mRBPome are also found in RNA granules isolated from *Drosophila* S2 cells. Seventeen of these proteins (CG6701, Hsc70Cb, Hsp83, Rack1, Nocte, Scu, Ncd, Hsc70-5, CG5726, Coro, Bic, Mtpα, CG7518, CG5787, CG8108, Thiolase, Tyf) were not previously annotated as RBPs and possess no known RBD. Thus, these 17 represent novel proteins that may directly interact with RNA within granules.

### Validation of RNA-binding activity of candidate RBPs

**Figure 3.**
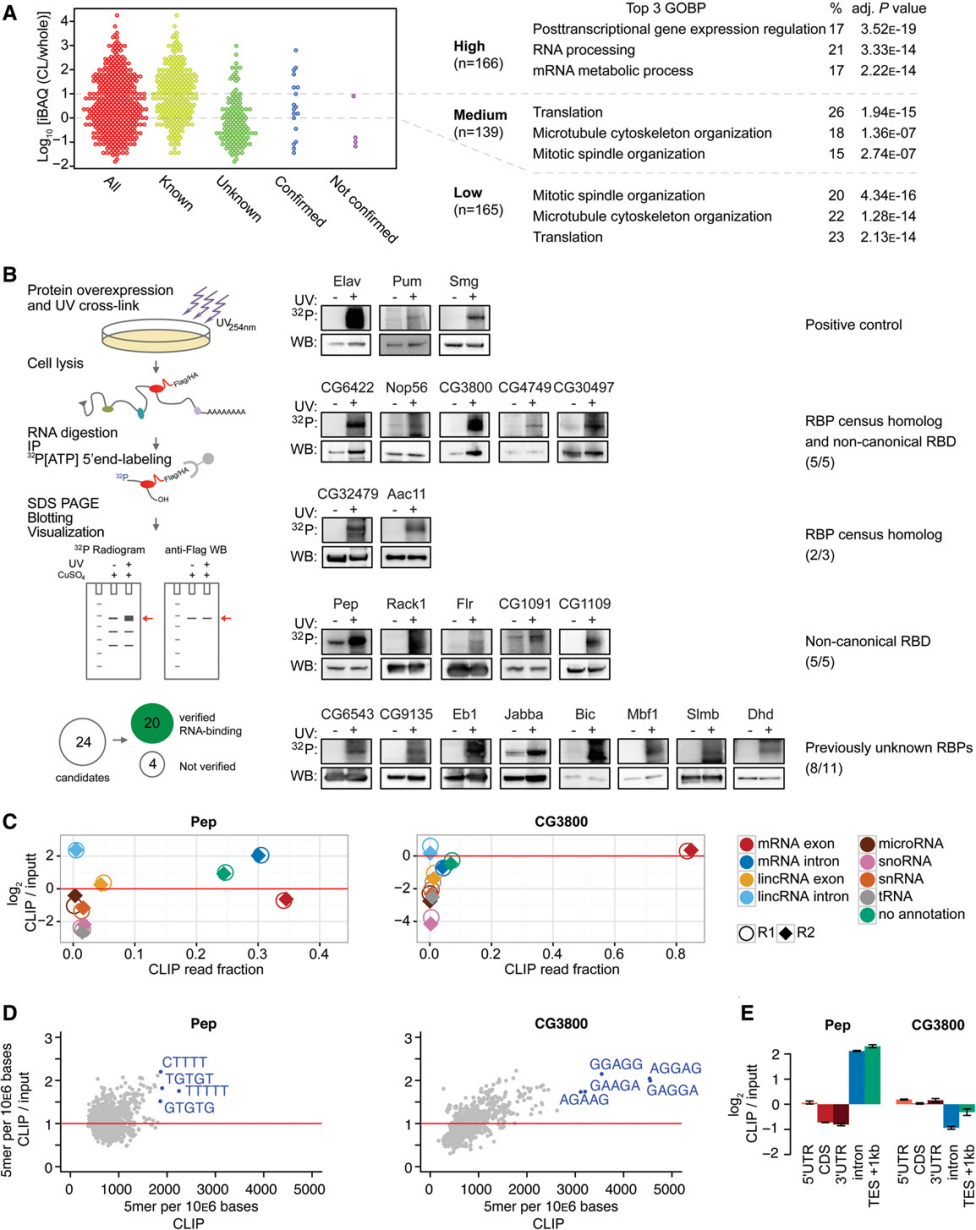
Validation of direct RNA interaction of candidate RBPs. (*A*) Protein enrichment (log_10_ iBAQ ratios of proteins in oligo[dT] precipitate versus whole-embryo proteins). Proteins are divided by enrichment score (high = >10-fold enrichment, middle = 1–10-fold enrichment, low = <1-fold enrichment) (six proteins [two validation candidates] missed whole embryo detection and could not be added here). The three most enriched GO-terms for biological processes (GOBP) for each category are shown on the *right*. Validation candidates were chosen throughout the enrichment ratio range, and were not annotated as RNA-binding in GOMF or having classical RBDs (except Pep). (*B*) Scheme describing the experimental approach. Epitope-tagged candidate RBPs were expressed in transiently or stably transfected *Drosophila* S2 cells. After 254-nm UV-crosslinking, cell lysis, and RNase digestion, the immunoprecipitated crosslinked protein-RNA complexes were radiolabeled by T4 PNK phosphorylation and separated by SDS-PAGE. ^32^P-autoradiogram and Western blot analysis of 254-nm UV-crosslinked (+) and noncrosslinked (−) complexes for each indicated RBP candidate are shown. Known RBPs (Elav, Pum, Smg), served as positive controls. RNA-signals were compared against FLAG-IP of crosslinked parental S2 cells to estimate nonspecific signal. RBP candidates Wech, GlyP, Hsc70Cb, and CG6287 could not be verified. (*C*) Enrichment of uniquely aligned CLIP sequencing reads relative to matched total RNA input for Pep and CG3800. *X*-axis: fraction of reads per million (RPM). *Y*-axis: log_2_-transformed RPM ratio of CLIP vs input samples. (Circles, replicate 1; diamonds, replicate 2.) (*D*) 5-mer enrichment of randomly sampled aligned RBP CLIP reads relative to matched inputs. *X*-axis: frequency of 5-mer in 10^6^ bases. *Y*-axis: 5-mer frequency ratio CLIP vs. input. (*E*) Enrichment analysis of aligned sequencing reads to mRNA subannotation categories. Sequencing reads were normalized to reads per kilo base per million (RPKM). *Y*-axis: log_2_-transformed ratio of annotation categories RPKMs in CLIP vs. input. (TES) transcription end site.

To quantify protein enrichment in precipitates relative to protein abundance in early embryos, we ranked all early fly mRBPome proteins by their protein iBAQ ratio of oligo(dT) precipitate and embryo proteome and divided the protein set into three enrichment classes (Fig. 3A). Although known RBPs exhibited on average higher enrichment, we encountered known RBPs throughout the entire ratio range (Fig. 3A). The early fly mRBPome was overall enriched for developmentally essential genes (Supplemental Fig. S3A; Supplemental Table S3), possibly related to their function as RBPs. However, the three enrichment classes held comparable proportions of essential genes (Supplemental Fig. S3B). GO-term analysis indicated that the highly enriched RBPs showed mostly RNA-processing terms, while the medium and low classified proteins were annotated with predominantly cytoskeleton-related and translational regulation functions (Fig. 3A).

We selected 24 candidate RBPs (eight from each enrichment class), which are currently not annotated as RBPs and do not contain classical RBDs (except Pep [Hamann et al. 1998]), for validation by crosslinking and immunoprecipitation (CLIP) (Ule et al. 2005). For 20 of the 24 candidate RBPs expressed in *Drosophila* S2 cells, we observed specific ^32^P-RNA signals size-matched to respective protein signals on corresponding Western blots (Fig. 3B). All candidates containing predicted noncanonical RBDs (10/10) bound directly to RNA. Among the 14 undescribed or homology-inferred RBP candidates, 10 showed RNA-binding by CLIP.

We performed CLIP-seq on Pep and CG3800 in *Drosophila* S2 cells to confirm that the majority of RBP-bound RNA fragments map to mRNA sequences. For both RBPs, 65%–85% of all reads mapped to primary or mature mRNA transcripts (Fig. 3C). At the same time, CLIP-seq samples of both RBPs were depleted for other RNA species (tRNA, snRNA, snoRNA, miRNA) relative to matched input samples. According to its postulated function in mRNA splicing, Pep-bound RNA fragments were strongly enriched for intronic sequences in both mRNA and lincRNA, although we did not observe distinct sequence specificity (Fig. 3D; Supplemental Fig. S3C). A considerable fraction of sequence reads aligned directly downstream from annotated transcription end sites (Fig. 3E), implying an additional role for Pep in binding to nascent RNA, likely during transcription up to transcription termination. In contrast, CG3800 bound mostly to mRNA exons (Fig. 3C,E) and showed clear preference for binding to GA-rich RNA sequences (Fig. 3D).

### RBPs and TFs show distinct expression dynamics in *Drosophila melanogaster* development

The transcriptome-wide determination of fly RBPs provides an opportunity to investigate expression dynamics of post-transcriptional regulators, using the assumption that RNA and protein levels are correlated in fly embryos (Supplemental Fig. S4A).

**Figure 4.**
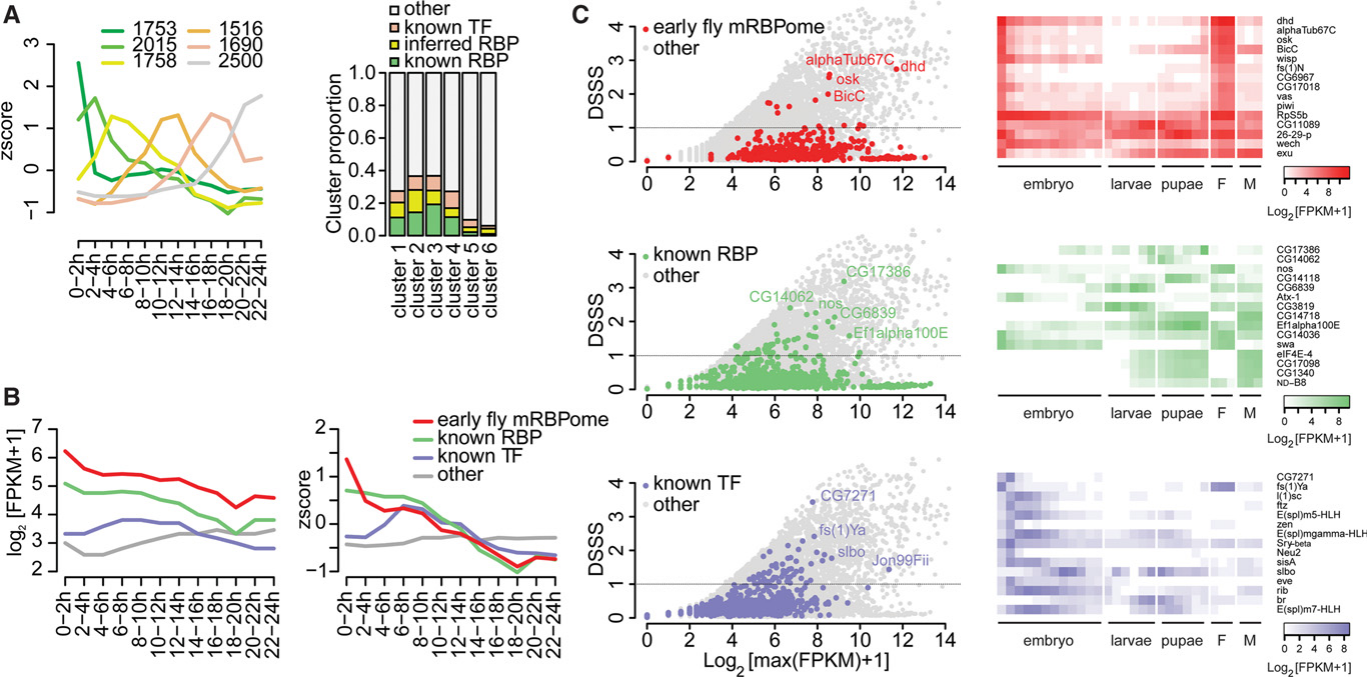
RBP expression in *Drosophila* development. (*A*) Clustering of *z*-score-transformed gene expression levels of protein-coding genes expressed during embryogenesis (0–24-h post egg laying) (*n* = 11,232) (Graveley et al. 2011) into six condensed, timely resolved clusters. (*Left*) Cluster median *z*-score-transformed expression values over time. Clusters sizes are indicated in order 1 through 6. (*Right*) Cluster proportions of all known RBP (harboring RBDs or RNA-binding GO annotation), inferred RBPs (as described in Fig. 2D), and known TFs (FlyTF.org). (*B*) Median of absolute expression values (log_2_[FPKM + 1]) and relative expression values (*z*-score) of the early embryo mRBPome without ribosomal proteins, known RBPs without ribosomal proteins, and known TFs over time during embryogenesis. (*C*) Developmental Stage Specificity Score (DSSS) for genes expressed during 30 developmental stages (Graveley et al. 2011) for all protein-coding genes (*n*=13,452). Scatterplot of DSSS relative to log_2_-transformed maximal expression levels for the indicated subsets (*left*) and heat map of log_2_-transformed FPKM + 1 expression values of the 15 most specific members of each subset.

We clustered transcript expression of all protein-coding genes in *Drosophila* embryogenesis, deriving at six temporally resolved clusters (Fig. 4A, left). RNAs encoding the early embryo proteome and mRBPome were overrepresented in the earlier clusters (Supplemental Fig. S4B). Maternal and early zygotic cluster 2 was especially rich in post-transcriptional gene regulation terms, while clusters 3 and 4 showed more general RNA processing and protein translation terms (Supplemental Table S2). Moreover, early clusters showed enrichment for GO-terms associated with structural components and RNA localization terms, whereas later clusters comprised genes involved in metabolic processes.

We asked whether the enrichment of RBPs in early clusters was due to the biased approach of mRNA interactome capture in 0–2-h fly embryos, or a general feature of *Drosophila* embryogenesis, and contrasted these findings to the corresponding patterns of transcription factors (TFs). Matching the distribution of known RBPs in the early fly mRBPome (Supplemental Fig. S4B), the first four clusters consisted of up to 18% of known RBPs (Fig. 4A, right). Later clusters were largely depleted of both RBPs and TFs. Analogous to other studies (Gerstberger et al. 2014; Kechavarzi and Janga 2014), we found that RBPs are more highly expressed compared to TFs (Fig. 4B, left). Relative RBP expression peaked throughout the first 8 h of embryogenesis, suggesting that post-transcriptional and translational regulation plays an important role during prezygotic and MZT stages (Fig. 4B, right). Previously unknown RBPs that exhibit lower enrichment showed higher gene expression specificity in the first 2 h of embryogenesis (Supplemental Fig. S4C,D). In contrast, TF expression peaked between the first zygotic waves and mid-embryogenesis (Fig. 4B). Thus, RBPs and TFs may divide embryogenesis into distinct temporal units, in which each of these two regulator classes dominates gene expression regulation.

Next, we analyzed the *Drosophila* transcriptome at 30 time points ranging from early embryos through adult female or male flies and calculated a Developmental Stage Specificity Score (DSSS) (see Methods). Most RBPs and TFs show no temporal specificity (Supplemental Fig. S4E). Only 14 (3%) genes of the early fly mRBPome, 39 (4.6%) known RBPs beyond the mRBPome, and 60 (8%) TFs showed DSSS ≥ 1, in contrast to 2717 (20%) of all protein-coding genes (Supplemental Fig. S4E,F). The highest scoring genes in the early fly mRBPome were specifically expressed in the first 2 h of embryogenesis and in adult females (Fig. 4C). Besides expected genes such as *oskar* and *piwi*, we found *dhd* to have the highest specificity score. Dhd is the fly homolog of human TXN and has been shown to alleviate dopaminergic neuron loss induced in a fly model for Parkinson's disease (Umeda-Kameyama et al. 2007). Dhd was not previously known to bind RNA.

Among known RBPs outside the early fly mRBPome, CG17386 (homologous to human LARP6) was specifically expressed near the end of the pupal stage (Fig. 4C). Four of the 15 genes most specifically expressed during larvae and pupae stages (CG14062, CG14118, CG6839, and CG3819) encode proteins with annotated endonuclease activity (Fig. 4C, right).

### Embryonic RBP transcript localization

**Figure 5.**
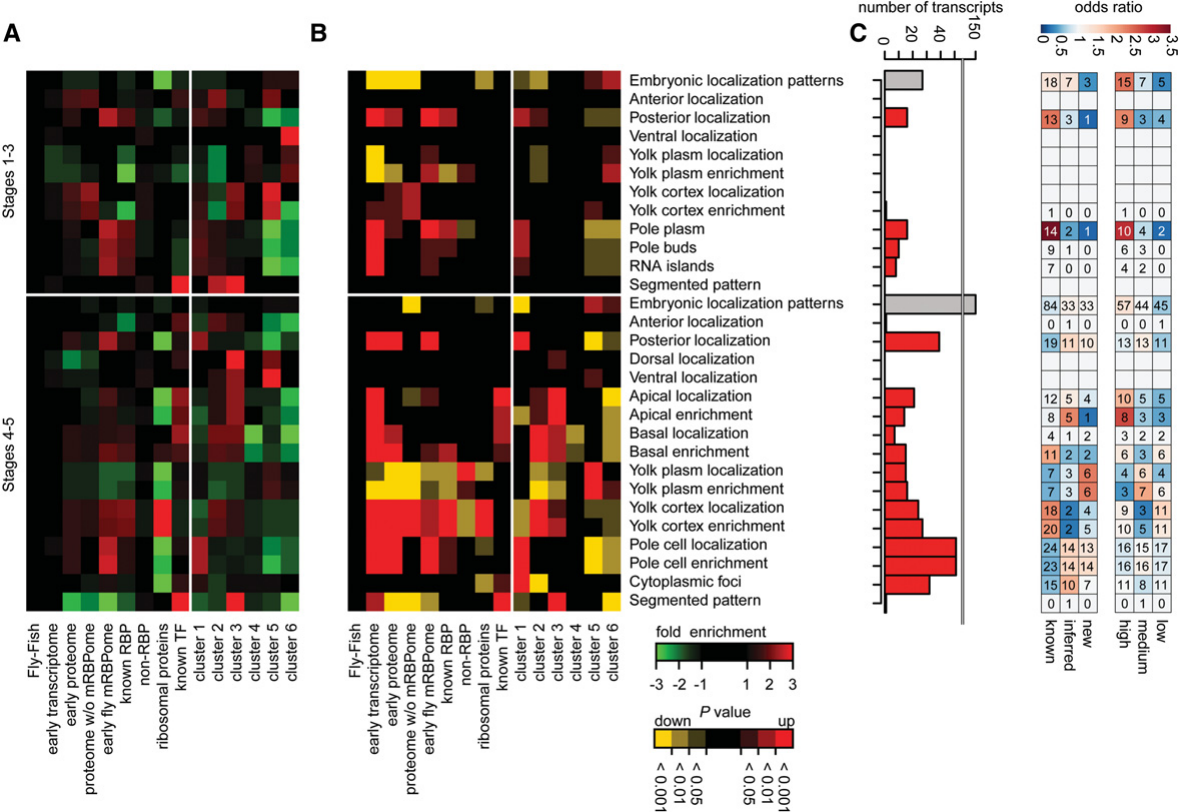
Localization of RBP-enocding mRNAs in early *Drosophila* embryos. RNA fluorescent in situ hybridization information from the Fly-FISH database (Lécuyer et al. 2007) was used to empirically calculate overrepresented transcript localization annotation terms by random sampling relative to the Fly-FISH database. Heat map indicating fold changes (*A*) and *P* values (*B*) for each subset for embryonic localization terms in embryonic developmental stages 1–3 and 4–5. Early transcriptome: protein coding genes expressed (FPKM > 0) in 0–2-h embryos; early proteome: proteins identified by whole 0–2-h embryo mass spectrometry; proteome without mRBPome: does not contain genes identified by mRBPome capture; known RBPs: RBPs selected by GO-term and RBD within the transcriptome excluding ribosomal proteins; non-RBP: transcriptome without known RBPs; ribosomal proteins: all ribosomal proteins; known TF: transcription factors from FlyTF.org database. Clusters as in Fig. 4A. Relative subset representation can be assessed in Supplemental Figure S5A. (*C*) The number of transcripts of the early fly mRBPome for each localization category. Gray bars = number for all transcripts with spatially restricted (≠ ubiquitous) embryonic localization terms within stages 1–3 and 4–5, respectively; red bars = number of transcripts in individual localization categories. Odds ratio of subcategories relative to the early fly mRBPome. Subcategories represent classification from Figures 2D and 3A.

Many known gene products controlling *Drosophila* development are restricted to specific regions of the early embryo (Lécuyer et al. 2007; Jambor et al. 2015). We asked whether mRNAs encoding the early embryo mRBPome are specifically localized using early embryo transcript localization data from Fly-FISH (Lécuyer et al. 2007), assuming mRNA localization partially reflects protein localization. RBP-coding transcripts were enriched in posterior regions of the early embryo (Fig. 5A,B; Supplemental Table S4), which differed from TF mRNA localization. Ribosomal proteins have been found to be enriched in primordial germ cells (Siddiqui et al. 2012), but mRNAs coding for ribosomal proteins did not contribute to the posterior enrichment observed for RBP transcripts. The observed RBP and TF transcript spatial enrichments were recapitulated for the six previously defined gene expression clusters (Fig. 5A,B; see also Fig. 4B).

In total, 28 (8%) and 150 (46%) transcripts of the early fly mRBPome present in the database (*n* = 326) showed spatially restricted embryonic localization patterns at stages 1–3 and stages 4–5, respectively (Fig. 5C). Fifteen transcripts showed restricted posterior localization from the onset of embryogenesis, including *oskar* (Supplemental Fig. S5B), while 38 RBP-encoding transcripts were enriched in posterior regions during later stages 4 to 5, including *exu*, *lost*, *pum*, and *piwi* mRNAs. RBP candidates Hsp83 and CG6967 showed similar localizations but have not been previously described as RBPs (Supplemental Fig. S5B). Among the posterior localized RBP-coding transcripts, we encountered a class of structural component-associated proteins including Flr, Top2, and BicD, which have been described to either bind directly to RNA or were found to be in RNP transport complexes (Supplemental Fig. S5B; Rzepecki and Fisher 2000; Bullock and Ish-Horowicz 2001; Dienstbier et al. 2009).

Two RBP candidates (For and CycT) showed striped expression patterns (Supplemental Fig. S5B). The protein For has been described to have cGMP-dependent serine/threonine kinase activity (Osborne et al. 1997). CycT, identified only by the cCL approach, harbors kinase activity (Lis et al. 2000) and is orthologous to the human RBP census members CCNT1 and 2.

Taken together, similar to complementary temporal expression patterns of RBPs and TFs, transcripts coding for RBPs and TFs also showed distinct spatial enrichments.

## Discussion

In this study, we describe a high confidence set of 476 poly(A)^+^ RBPs in early *D. melanogaster* embryos. We used two complementary UV-crosslinking approaches: conventional UV-crosslinking, and the use of transgene-supplied UPRT, which allows for 4SU incorporation before UV-crosslinking at a longer wavelength.

The two complementary UV-CL methods identified different numbers of proteins in oligo(dT)-purified RNA-protein complexes. Specifically, we did not reach detection saturation in the PAR-CL experiment. The disparity in protein abundance between the two approaches is most likely caused by differences in the amount of embryos used (cCL: 6g, PAR-CL: 2.6g), rather than insufficient 4SU labeling or differences in crosslinking efficiency, though the latter cannot be ruled out.

Despite the difference in protein number, both approaches yielded comparable protein enrichments (Supplemental Fig. S1D) as previously observed for a cCL- and PAR-CL-based mRNA interactome capture study (Castello et al. 2012). Many known RBPs were identified, but several RBPs such as Swallow and Nanos (Chao et al. 1991; Wharton and Struhl 1991; Sonoda and Wharton 1999; Schnorrer et al. 2000), which specifically bind maternal transcripts, were not detected by either method, likely as a result of finite sensitivity and dynamic range of mass spectrometry-based proteomics.

In spite of these limitations, we identified a conservative set of 476 poly(A)^+^ binding proteins, of which 99 are new RBP candidates previously undescribed in terms of direct RNA interactions (Fig. 2D). Given that 203 of the identified proteins did not have a known RBD and that we could confirm RNA-binding for 10 out of 14 proteins with no prior knowledge about RBDs (Fig. 3B), our results suggest that mRBPome capture is suitable for unbiased identification of novel RBPs in vivo (see also Supplemental Fig. S2A).

There is an apparent enrichment of structural component-associated factors within the early fly mRBPome. Well-studied RBPs like Staufen and bicoid stability factor (BSF) are known to mediate *bicoid* RNA localization (Ephrussi et al. 1991; Ferrandon et al. 1994; Mancebo et al. 2001). Although known to be involved in *bicoid* transcript localization, there was so far no evidence for direct RNA-binding for the microtubule-associated protein mini spindles, Msps (Moon and Hazelrigg 2004). Bicoid protein has been implicated in RNA-binding (Rivera-Pomar et al. 1996) but was not detected in this mRBPome capture study. Similar to *bicoid* RNA localization factors, the early fly mRBPome comprises Oskar and 18 proteins involved in *oskar* localization.

Interestingly, RBP-encoding mRNAs show enrichments in posterior regions (Fig. 5A). Examples include Top2, Flr, and BicD. Top2 was previously shown to directly interact with RNA (Rzepecki and Fisher 2000). Flr binds actin and contains a WD40 domain, which is usually implicated in protein-protein interactions but can also have potential RNA-binding properties (Lau et al. 2009; Stirnimann et al. 2010; Kwon et al. 2013; Loedige et al. 2015). Correspondingly, we found RNA crosslinking evidence for about 30 out of 95 WD40 domain-harboring proteins of the early fly proteome (Supplemental Fig. S2B) and specifically confirmed RNA-binding for Flr and two other WD40 proteins (CG1109, Rack1) (Fig. 3B). Moreover, we identified BicD, a protein with noncanonical RBDs in the mRNA-bound proteome. BicD is a dynein adaptor protein anchoring the RNA-binding protein Egl (Dienstbier et al. 2009) to dynein motors (Bullock and Ish-Horowicz 2001; Liu et al. 2013). The enrichment of BicD in oligo(dT) precipitate suggests that the early fly mRBPome harbors previously uncharacterized RBPs directing transcript localization during early embryogenesis.

A number of well-characterized RBPs such as Tudor, Piwi, Oskar, Vasa, and Aubergine have been described to exhibit spatial-restricted function in the posterior embryo (Hay et al. 1988; Bardsley et al. 1993; Breitwieser et al. 1996; Harris and Macdonald 2001; Megosh et al. 2006). A recent study reported enrichment of 62 proteins in sorted primordial germ cells in early *Drosophila* embryos (Siddiqui et al. 2012). Of these, 18 (29%) proteins were found in the early fly mRBPome (14 known RBPs; four previously unknown: Pp1-87B, CG7920, CG8036, and Mcm7). However, only three of these were supported by Fly-FISH transcript localization data (Oskar, Piwi, and CG8036), suggesting either an incomplete picture of transcript localization or that there are various mechanisms of protein localization besides transcript localization and local translation that contribute to RBP enrichment in the posterior embryo.

While this study was under review, two mRBPome capture studies covering yeast, *Caenorhabditis elegans*, and human HuH-7 hepatoma cells have been published, (Fig. 2E; Beckmann et al. 2015; Matia-González et al. 2015). Remarkably, 362 proteins of our 476 high-confidence RBPs and 47 of our 99 novel RBP candidates were also detected in at least one other study (Supplemental Fig. S6A,B).

In summary, our study emphasizes the importance of experimental methods for the identification of novel RBPs in complex model organisms. Our data set comprises nearly 100 RNA-interacting proteins with noncanonical RNA-binding domains as potentially novel post-transcriptional regulators. The high sequence identity of RBPs suggests that the regulatory function of these proteins is deeply conserved and therefore not only of interest to *Drosophila* developmental biologists, but to scientists working on early embryogenesis in other model organisms.

## Methods

### UV-crosslinking of *D. melanogaster* embryos

Adult *yw* flies were fed with standard yeast paste and adult *UPRT*-expressing X490 flies with yeast paste containing 4-thiouracil. Staged 0–2-h old embryos were collected for irradiation with UV at 254- and 365-nm wavelength, respectively. During UV-irradiation, embryos were constantly chilled on ice to prevent further development. Embryos were washed with deionized water and dechorionated with bleach before freezing and storing at −80°C. Detailed information on fly strains used, metabolic labeling of *D. melanogaster* embryo RNA with 4-thiouridine, 4-thiouridine incorporation assay, and crosslinking procedure is provided in Supplemental Methods.

### Isolation of mRNA-interacting proteins in *D. melanogaster* embryos

Frozen embryos were thawed on ice and lysed in 10–20 mL lysis/binding buffer per gram embryo weight (100 mM Tris HCl, pH 7.5, 500 mM LiCl, 10 mM EDTA, pH 8.0, 1% (w/v) lithium-dodecylsulfate, 5 mM DTT, Complete Mini EDTA-free protease inhibitor [Roche]). For a typical proteomics experiment, 25 mL oligo(dT) Dynabeads (NEB) (bead suspension volume in original storage buffer) were added to embryo extract prepared from 2500 mg (X490) and 6000 mg (*yw*) embryos. For each proteomics experiment, the same amount of nonirradiated embryos was processed in parallel. The oligo(dT)-precipitation was performed essentially as described in Baltz et al. (2012) with the following modifications: The number of washing steps was increased (4× with lysis/binding buffer and 6× with NP40 washing buffer [50 mM Tris HCl, pH 7.5, 140 mM LiCl, 2 mM EDTA, pH 8.0, 0.5% NP40, 0.5 mM DTT]) and after elution, the RNA was digested by incubation with RNase I at 25 U/mL and benzonase (62.5 U/mL) for 3 h at 37°C in elution buffer containing 1 mM MgCl_2_, and stored at −80°C for further analysis.

### Mass spectrometry sample preparation and analysis

Detailed description provided in Supplemental Methods.

### Validation of RNA-binding activity of novel RBP candidates

Candidate RBPs have been selected based on the mean (UV 254 nm and UV 365 nm) mass spectrometry iBAQ ratio between oligo(dT) pull-down samples (pull-down) and whole early embryo proteome samples (whole) (iBAQ intensities [pull-down/whole]; high = >10, medium = 1–10, low = <1; eight candidates from each enrichment class). Plasmids allowing expression of selected candidate RBPs as C-terminal FLAG fusions were obtained from the *Drosophila* Genome Research Centre (DGRC) (Supplemental Table S5). RNA interaction validation experiments (Kwon et al. 2013) were conducted in either transient transfected cells or stable S2 cells. Briefly, non- and UV254-nm-crosslinked transfected S2 cells were lysed, treated with RNase I and DNase, and RBP-FLAG proteins immunoprecipitated using an anti-FLAG antibody coupled to Protein G Dynabeads. Crosslinked RNA in protein-RNA complexes was radiolabeled with γ-^32^P-ATP using T4 polynucleotide kinase. Protein-RNA complexes were separated on SDS-PAGE, blotted onto nitrocellulose, and probed with anti-FLAG antibody by Western analysis for protein and Phosphorimager for radioactivity.

Detailed description of procedure to validate validation of RNA-binding activity is described in Supplemental Methods.

### CLIP-seq library preparation and CLIP-seq data analysis

A detailed description of CLIP-seq cDNA library preparation, data processing, and CLIP-seq specific analysis are provided in Supplemental Methods.

### Embryonic gene expression analysis

mRNA expression levels during *Drosophila melanogaster* development (embryo to adulthood, modENCODE [Graveley et al. 2011]) were precomputed and extracted from FlyBase release FB2015-02. The expression data were reduced to only protein-coding genes (CG annotated transcripts, source FlyBase). Proteomic data and transcriptomic data were matched by FBgn. Transcriptome categories were based on: known RBPs: list of proteins based GO-term as RNA-binding, Pfam RNA-binding domain or orthologous protein to human RBP census (Gerstberger et al. 2014); ribosomal proteins: list of proteins based on gene name and RNA-binding domain; proteins with RNA-related GO-terms; transcription factors: list of all experimentally validated and predicted TFs (www.flytf.org; release Feb. 2015). The GO-term based categorization, as well as the Pfam protein domain-based categorization, were adopted from Gerstberger et al. (2014).

For depicting transcript abundance, log_2_-transformed precomputed FPKM values were taken. A gene was considered to be expressed if FPKM > 0 for a given sample. Gene expression profiles were depicted by genewise *z*-score transformation of FPKM + 1 across all embryonic time points (0–24 h).

### mRBPome feature analysis

Analyses of gene set enrichment, isoelectric point, conservation, protein domain enrichment, protein disorder, and sequence complexity as well as overlap with human RBP census are described in Supplemental Methods.

### Gene expression clustering

The *z*-score-transformed developmental transcriptome was clustered into six clusters using the bioconductor R package mfuzz (Kumar and E Futschik 2007). (The optimal number of clusters was estimated with 10 clusters. Due to the small number of RBP candidates, we reduced the number to six clusters.) The cluster hardness was estimated to be optimal at approximately m = 1.23. RBP candidate cluster association was estimated from embryonic transcriptome clusters. RBP candidate cluster enrichment was calculated in two steps using Fisher's exact testing. Since the mass spectrometry protein quantification showed enrichment of higher-expressed genes, we first calculated cluster enrichment for the early embryo proteome and used this to estimate the mRBPome cluster enrichment.

### Calculating the developmental stage specificity score

The DSSS was calculated as previously described (Gerstberger et al. 2014). We used the precomputed expression data for all 30 developmental stages of the modENCODE fly developmental time course (see above). A total of 13,452 protein-coding genes with at least one sample with FPKM > 0 were considered for this analysis. We defined the DSSS as the deviation from a uniform expression across all developmental stages. DSSS takes the logarithm of the number of stages minus the Shannon entropy of the expression values for each gene:

$$\text{DSSS}=H_{\max}-H_{\mathrm{obs}}=\log_{2}(N)-\left(-\sum_{i=1}^{N}[\;p_{i}x{\;\log}_{2}(p_{i})]\right).$$

*p*_*i*_ is the relative frequency,

$$p_{i}=\frac{x_{i}}{\sum_{i=1}^{N}x_{i}}.$$

*x*_i_ is the FPKM + 1 expression level for gene *x* in tissue *i. H*_max_ is the maximal possible entropy. *H*_obs_ is the observed entropy. *N* is number of developmental stages; here, 30.

Dependent on the number of time points, the DSSS ranged from 0 for expression throughout all time points to 5 for highly time point-specific expression.

### Transcript localization information

Early embryo transcript localization data was retrieved from the Fly-FISH database (http://fly-fish.ccbr.utoronto.ca/; release June 2014) (Lécuyer et al. 2007). The gene identifiers have been matched to the FB2015-02 release. Genes with multiple entries have been aggregated into one entry bearing all localization information. Transcripts have been categorized based on functional annotations (cf. *Embryonic gene expression analysis*). Four thousand seven hundred sixty-seven protein coding genes had annotated information for at least one stage in the original data set.

In order to test for enrichment of specific embryonic transcript localization terms, we quantified the number of transcripts for each localization term for each subset and compared these against random sampling of all transcripts with localization annotation in the Fly-FISH database (*n* = 4767). The fold change for each category was calculated relative to the expected number of localized transcripts based on the distribution in the entire Fly-FISH database. The statistical significance was estimated by random sampling from the entire Fly-FISH database for the respective category size. The empirical *P* value depicts how often the number of transcripts per individual localization term was reached by chance in 1000 random samples. The odds ratio relative to the early fly mRBPome was calculated using R package GeneOverlaps v1.6.0 (http://shenlab-sinai.github.io/shenlab-sinai/).

All FISH images shown were extracted from (http://fly-fish.ccbr.utoronto.ca/), displaying the selected representative image.

## Data access

The mass spectrometry raw data from this study have been submitted to the ProteomeXchange (http://www.proteomexchange.org) under the data set identifier PXD002992. The CLIP-seq data have been submitted to the NCBI Gene Expression Omnibus (GEO; http://www.ncbi.nlm.nih.gov/geo/) under accession number GSE78237.
